# Confocal laser endomicroscopy for upper tract urothelial carcinoma: validation of the proposed criteria and proposal of a scoring system for real-time tumor grading

**DOI:** 10.1007/s00345-019-02646-5

**Published:** 2019-01-25

**Authors:** J. E. Freund, E. I. M. L. Liem, C. D. Savci-Heijink, J. Baard, G. M. Kamphuis, J. J. M. C. H. de la Rosette, D. M. de Bruin

**Affiliations:** 1 Department of Urology, Amsterdam UMC, University of Amsterdam, Amsterdam, The Netherlands; 2 Department of Pathology, Amsterdam UMC, University of Amsterdam, Amsterdam, The Netherlands; 3 Department of Urology, Istanbul Medipol University, Istanbul, Turkey; 4 Amsterdam UMC, University of Amsterdam, Amsterdam, The Netherlands; 5 Department of Biomedical Engineering and Physics, Amsterdam UMC, University of Amsterdam, Amsterdam, The Netherlands

**Keywords:** Histologic grading, Confocal laser endomicroscopy, CLE, Optical imaging, Urothelial carcinoma of the upper urinary tract, UTUC

## Abstract

**Purpose:**

Confocal laser endomicroscopy (CLE) is a fluorescence-based fiber-optic imaging technique with the potential for intraoperative grading of upper tract urothelial carcinoma (UTUC). This study aims to (1) investigate the prevalence of the previously proposed CLE criteria for bladder cancer in papillary UTUC, (2) estimate the diagnostic value of CLE for UTUC grading and (3) propose a scoring system for a more quantifiable approach of CLE-based grading of UTUC.

**Materials and methods:**

Ureteroscopic CLE was performed in patients with UTUC. Following CLE imaging, co-localized biopsies were taken for histopathologic comparison. Postoperatively, two blinded raters assessed the CLE images.

**Results:**

Fifty-three papillary UTUCs (34 low grade and 19 high grade) were imaged with CLE in 36 patients. All the previously described CLE criteria were identifiable in varying proportions. After excluding 10 non-diagnostic recordings (5 low grade and 5 high grade) due to insufficient image quality, the histopathologic grade was correctly identified with CLE in 26 low-grade UTUCs (90%) and in 12 high-grade UTUCs (86%). The most prevalent CLE criteria with the highest diagnostic potential were cellular organization, morphology and cohesiveness of cells. A scoring system was proposed with these criteria, which yielded similar diagnostic accuracies.

**Conclusions:**

Based on the previously proposed criteria, CLE enables accurate grading of papillary UTUC at a non-diagnostic rate of 19%. The most prevalent CLE criteria with the highest diagnostic potential for grading of papillary UTUC are cellular organization, morphology and cohesiveness of cells. The proposed scoring system may simplify the assessment of CLE images for UTUC grading but external validation is required.

## Introduction

The oncologic effectiveness of kidney-sparing treatment for upper tract urothelial carcinoma (UTUC) can only be warranted in selected patients [1, 2]. Risk stratification of UTUC has, therefore, become an essential step in the diagnostic pathway [3]. Endoscopic laser ablation is the treatment of choice in low-risk UTUC, while radical surgical resection is indicated in high-risk cases [2].

The histopathologic tumor grade is a key factor in the risk stratification of UTUC. Consequently, the need for tumor grade identification has augmented the importance of ureteroscopic biopsies. Real-time intraoperative risk stratification by histopathologic assessment is, however, lacking in the current diagnostic workup. Additionally, in 10–40% of ureteroscopic biopsies, the histopathologic grade is discordant with the tumor grade from surgical resection specimens [4–8]. Moreover, the non-diagnostic yield of ureteroscopic biopsies for UTUC grading ranges from 10 to 20% [4–6, 8].

Confocal laser endomicroscopy (CLE) is a fluorescence-based fiber-optic imaging technique that has been investigated for real-time differentiation of urothelial carcinoma (UC). These investigations have resulted in the proposal of CLE criteria for UC grading in the bladder and the upper tract [9–11]. Despite promising feasibility studies in the upper tract, the proposed CLE criteria have only been validated for urothelial carcinoma of the bladder (UCB) [12–14]. With regard to the similarity in histology of UCB and UTUC, identical CLE criteria are anticipated [15]. However, CLE imaging in the upper urinary tract requires the use of a smaller CLE probe than for cystoscopic imaging. We hypothesize that the smaller field of view, the larger depth of the confocal plane and the reduced optical resolution of the smaller ureteroscopic CLE probe influence the visual appearance of UTUC and hence the prevalence of the proposed CLE criteria [14, 16]. As a result, validation of the proposed CLE criteria for UTUC is required.

The first objective of this study is to identify the prevalence of the proposed CLE criteria for UCB in papillary UTUC. Secondly, the diagnostic accuracy of CLE for UTUC grading, including inter-rater agreement analysis, is evaluated. Thirdly, based on the CLE criteria with the highest diagnostic potential, we aim to propose a scoring system for a more quantifiable approach for CLE-based grading of UTUC.

## Materials and methods

### Study design

The study design was in line with the IDEAL stage 2b recommendations and approved by the institutional review board [17]. The study was registered at the Dutch Central Committee on Research involving Human Subjects (NL52989.018.16) and at Clinicaltrials.gov (NCT03013920). This prospective clinical trial was carried out as previously described and conducted according to the guidelines of good clinical practice [18].

### Patients

Adult patients, planned for diagnostic ureteroscopy (URS) due to the suspicion of UTUC or for follow-up after kidney-sparing treatment in the Amsterdam University Medical Centers, location AMC, were eligible for this study. Exclusion criteria were fluorescein allergy and pregnancy. Written informed consent was obtained from all the participants.

After inclusion, patients could be disqualified for the study due to the absence of visible lesions during URS. Furthermore, tumors could be disqualified from the study due to local recurrence at the same location as imaged during prior study participation.

### Study procedure

The study procedure was conducted as previously reported [18]. In short, if a suspect upper tract lesion was visualized during URS, CLE imaging of this lesion was performed. In case of multifocality, the best accessible lesion was imaged. The 2.7 Fr Uroflex-B probe, interfaced with the 488-nm laser system, was used for CLE imaging. This multifiber-based probe yields a field of view of 320 µm, a lateral resolution of 3.5 µm in a confocal plane from 40- to 70-µm imaging depth.

CLE imaging was performed by experienced endourologists who had previously used CLE for UCB imaging [14]. Via the ureteroscope’s working channel, 0.5 mL of 2.5% fluorescein solution was injected onto the region of interest for CLE imaging. The Uroflex-B CLE probe was then introduced via the working channel of the semirigid or flexible ureteroscope and was placed in direct contact with the tissue of interest [19]. At least two CLE recordings of 1 min (8–12 frames/s) were obtained per lesion. Subsequently, a ureteroscopic biopsy was taken from the imaged lesion. Histopathologic workup and analysis were performed according to standard clinical protocol by a uropathologist (CDS), blinded for CLE images. UTUCs were graded according to the WHO 2004 classification [15]. The histopathologic grade from the tissue biopsies was used as the reference test.

### CLE image assessment

The presence of the proposed CLE criteria (papillary configuration, organization of cells, cohesiveness of cells, cellular morphology, definition of cell borders, vasculature and polarity) was assessed by two experienced CLE raters (JEF and CDS). Both raters were trained with a CLE training module and the assessment of CLE recordings of UCB [10, 18]. After a washout time of at least 3 months after obtaining CLE recordings, both raters, blinded to any clinical information and histopathology, evaluated the CLE recordings individually offline with the Cellvizio^®^ Viewer software (Mauna Kea Technologies, Paris, France). Based on the UCB CLE criteria, the observers graded the recordings as low-grade or high-grade UTUC. In case of insufficient image quality, the CLE recording was considered as non-diagnostic.

After individual assessment, consensus for the CLE criteria and the CLE-based grading was reached for each lesion. The analysis of the prevalence of CLE features and the comparison of the CLE-based grading with the histopathology of the biopsied tissue were performed with the results of the consensus.

### Sample size and statistical analysis

The sample size was based on the IDEAL recommendations for explorative studies and is in line with previously published CLE studies on UCB [11, 14, 17]. Flat lesions were excluded for the final analysis.

For the first objective, descriptive statistics were used to analyze the prevalence of CLE features for UTUC grade.

For the second objective, the diagnostic accuracy was assessed by estimating the sensitivity, specificity, positive predictive value (PPV) and negative predictive value (NPV) of each individual CLE criterion and overall CLE-based grading. These estimations were calculated in comparison with the histopathologic grade by 2 × 2 tables for the cohort with and without the non-diagnostic CLE yield. The non-diagnostic yield was defined as the proportion of lesions with non-diagnostic CLE recordings. The inter-rater agreement for the CLE criteria and CLE-based grading was determined as percentage agreement between the two raters. A threshold of minimally 80% agreement was considered as acceptable agreement [20].

Third, the CLE criteria with the highest prevalence, PPV and NPV and with both sensitivity and specificity of greater than 50% were selected for the proposal of a scoring system for UTUC grading. Besides different combinations of CLE criteria, different allocations of points for the presence of high-grade CLE features were evaluated for the scoring systems. High-grade features could score either two or three points while low-grade features scored 1 point and undefined features were allocated 0 points. The diagnostic ability of the different scoring systems was evaluated by receiver operating curve (ROC) analysis with area under the curve (AUC) testing against the null hypothesis (AUC = 0.5). Additionally, DeLong testing was performed for pairwise comparison of the AUC of the different scoring systems. Validation of the proposed scoring systems could not be performed due to the limited amount of data.

Statistical analyses were performed using SPSS Statistics version 24 and MedCalc V18.6.

## Results

### Patient and tumor characteristics

**Fig. 1:**
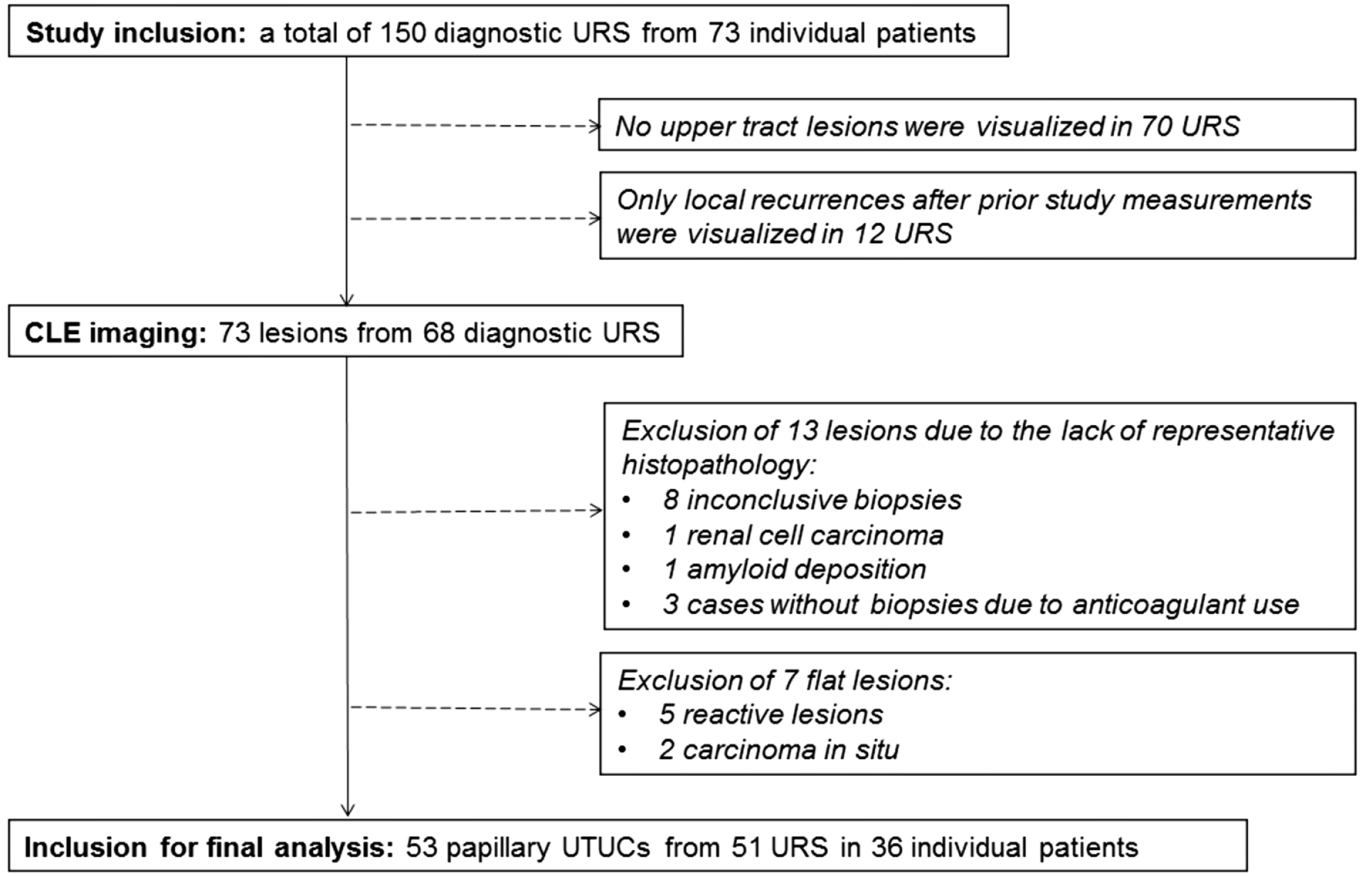
Flow diagram of inclusion for final analysis (*URS* ureteroscopy, *UTUC* upper tract urothelial carcinoma)

From August 2016 until March 2018, a total of 150 procedures in 73 individual patients were included. CLE imaging was performed in 68 procedures, in which upper urinary tract lesions were visualized. The CLE recordings of 53 papillary UTUCs from 51 procedures in 36 patients were included in the final analysis (Fig. 1). Patient and tumor characteristics are presented in Table 1.

**Table 1:** Patient and tumor characteristics

A. Patient characteristics	*n* = 36
Age in years, mean (sd)	70 (11)
Gender, *n* (%)	
Female	11 (31)
Male	25 (69)
Prior UTUC, *n* (%)	18 (50)

B. Tumor characteristics	*n* = 53
Laterality of disease, *n* (%)	
Left	27 (51)
Right	26 (49)
Tumor location, *n* (%)	
Ureter	23 (43)
Pyelocalyceal system	30 (57)
Tumor diameter in mm, median (range)	8 (2–100)
Tumor grade (WHO 2004), *n* (%)	
Low-grade	34 (64)
High-grade	19 (36)

*sd* standard deviation, *UTUC* upper tract urothelial carcinoma

### Prevalence of CLE criteria

**Fig. 2:**
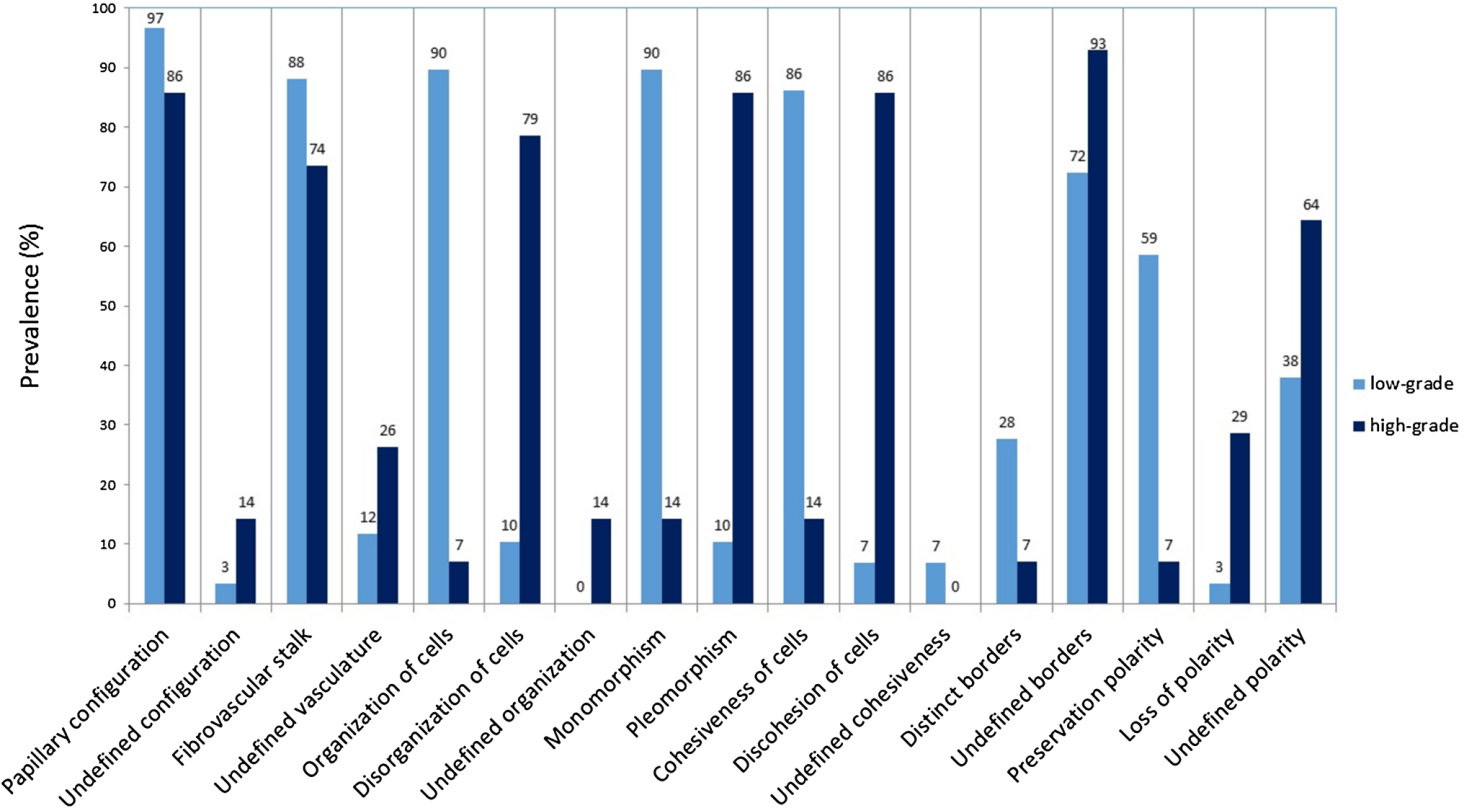
Prevalence of confocal laser endomicroscopy features in low-grade and high-grade upper tract urothelial carcinomas

**Fig. 3:**
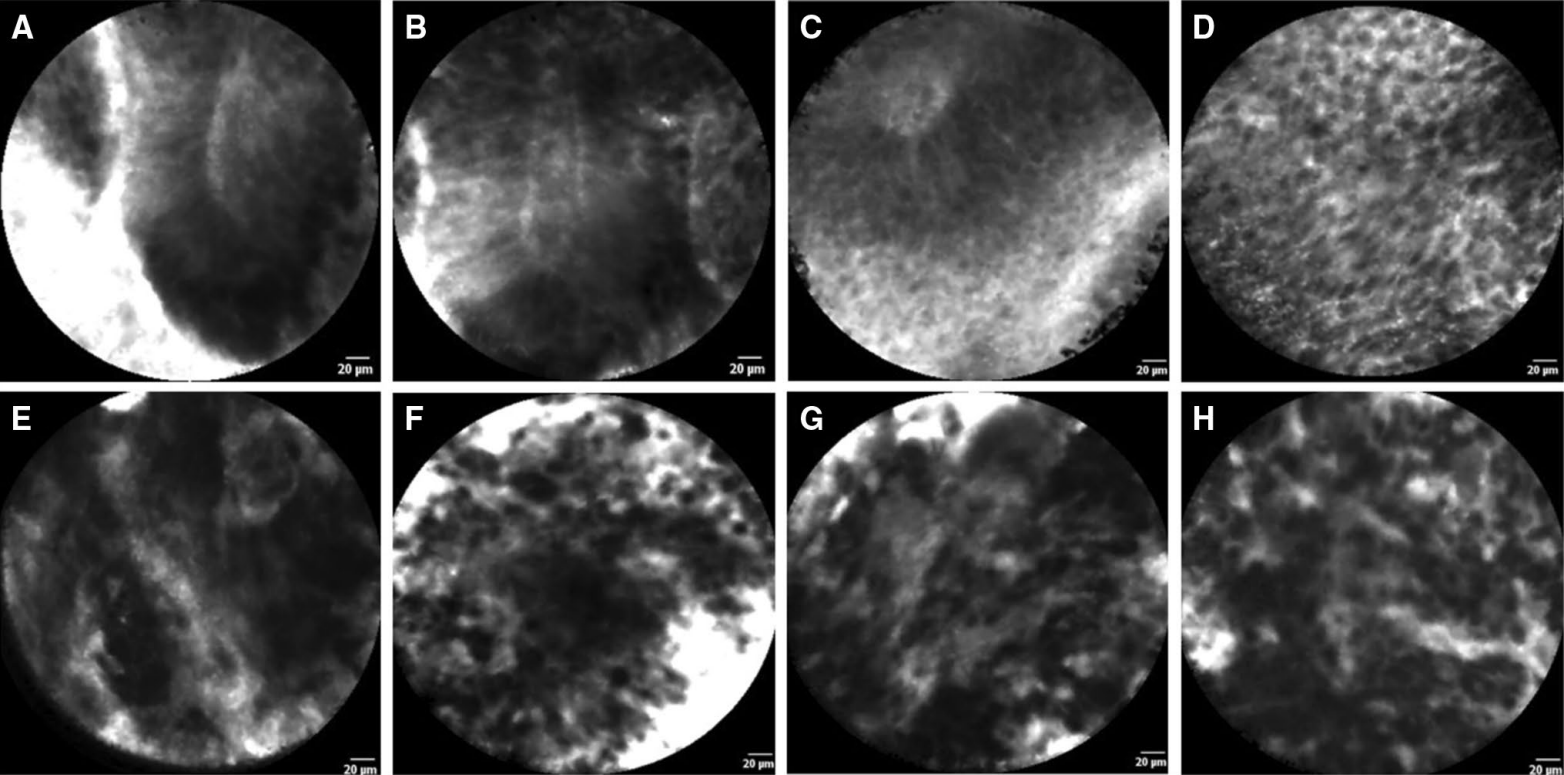
In vivo confocal laser endomicroscopy images of low-grade (**a**–**d**) and high-grade (**e**–**h**) upper urinary tract urothelial carcinomas: **a**, **b** cohesive, papillary configuration with fibrovascular stalk; **c** fibrovascular stalk with preservation of polarity; **d** cohesive and organized cellular architecture of monomorphic cells with distinct cell borders; **e** fibrovascular stalk and disorganized cellular architecture; **f** discohesive pleomorphic cells; **g**, **h** disorganization of pleomorphic cells

All the previously described CLE criteria were identifiable in varying proportions of low-grade and high-grade UTUCs. The prevalence of the CLE criteria per tumor grade of all 53 papillary UTUCs are presented in Fig. 2. The most prevalent CLE features between low-grade and high-grade UTUCs were: organization versus disorganization of the cellular architecture; monomorphism versus pleomorphism of cells; and cohesiveness versus discohesion of cells. Representative examples of the identified CLE features are presented in Fig. 3.

### Diagnostic accuracy estimates

For the complete cohort, the assessment of all the 7 CLE criteria resulted in a correct grade prediction in 38 of the 53 papillary UTUCs (72%, 95% CI 58–83%). The sensitivity for low-grade UTUC of the complete cohort was 77% (95% CI 59–89%) with a specificity of 63% (95% CI 38–84%). In five low-grade (15%) and five high-grade (26%) tumors, the CLE recordings were rated as non-diagnostic due to insufficient CLE image quality for CLE feature identification. When excluding the non-diagnostic recordings, assessment of all the CLE criteria resulted in a correct grade prediction in 38 of the 43 UTUCs (88%, 95% CI 57–92%) with a sensitivity for low-grade UTUC of 90% (95% CI 73–98%) and a specificity of 86% (95% CI 78–98%). The sensitivity, specificity, PPV, and NPV of the individual CLE criteria are presented in Table 2. The CLE criteria of cellular organization, cellular morphology and cellular cohesiveness achieved the highest diagnostic accuracy estimates.

**Table 2:** Inter-rater percentage agreement for each CLE feature and overall CLE-based grading, and estimations for the sensitivity/specificity and the positive predictive value/negative predictive value with regard to low-grade UTUC

CLE criteria	CLE feature variables	Calculations with the total cohort including (*n* = 53)					Calculations with the diagnostic cases only (*n*= 43)				
		Inter-rater percentage agreement	Sensitivity in % [CI]	Specificity in % [CI]	PPV in % [CI]	NPV in % [CI]	Inter-rater percentage agreement	Sensitivity in % [CI]	Specificity in % [CI]	PPV in % [CI]	NPV in % [CI]
Papillary configuration	Papillary/not papillary/undefined	91	91 [76–98]	21 [6–46]	67 [62–73]	57 [52–78]	95	97 [82–100]	14 [2–43]	70 [65–75]	67 [17–95]
Vasculature	Fibrovascular stalk/large vessels/undefined	87	85 [69–95]	16 [3–40]	64 [59–70]	38 [14–69]	86	100 [88–100]	21 [5–51]	73 [67–78]	100 [33–100]
Cellular organization	Organized/disorganized/undefined	93	77 [59–89]	58 [34–80]	76 [65–85]	58 [40–74]	93	90 [73–98]	79 [49–95]	90 [76–96]	79 [59–92]
Cellular morphology	Monomorphic/pleomorphic/undefined	91	77 [59–89]	63 [38–84]	79 [67–87]	60 [43–75]	91	90 [73–98]	86 [57–98]	93 [78–98]	80 [57–92]
Cellular cohesiveness	Cohesive/discohesive/undefined	70	76 [59–89]	74 [49–91]	84 [71–92]	64 [47–77]	74	86 [68–96]	86 [57–98]	93 [77–98]	75 [54–88]
Cellular polarity	Polarity/loss of polarity/undefined	83	50 [32–68]	21 [6–46]	64 [50–77]	19 [8–37]	79	59 [39–76]	29 [8–58]	63 [52–73]	25 [12–46]
Definition of borders	Distinct/undefined	83	24 [11–41]	95 [74–100]	89 [52–98]	41 [36–46]	79	28 [13–47]	93 [66–100]	89 [53–98]	38 [32–45]
CLE-based grading	Low grade/high grade/non-diagnostic	93	77 [59–89]	63 [38–84]	79 [67–87]	60 [43–75]	93	90 [73–98]	86 [57–98]	93 [78–98]	80 [57–92]

*CI* confidence interval, *CLE* confocal laser endomicroscopy, *NPV* negative predictive value, *PPV* positive predictive value

### Inter-rater agreement

The inter-rater percentage agreement for CLE criteria assessment and CLE-based grading between the two raters are presented in Table 2. The inter-rater percentage agreement was acceptable for all the CLE criteria except for cellular cohesiveness [20].

### Proposal of a CLE-based scoring system for UTUC grading

Two scoring systems with an allocation of either 2 or 3 points for high-grade features were proposed for the CLE criteria of cellular organization and cellular morphology (labeled as ‘2 criteria—2 points’ and ‘2 criteria—3 points’). Two additional scoring systems were proposed by adding cellular cohesiveness to the above-mentioned systems (labeled as ‘3 criteria—2 points’ and ‘3 criteria—3 points’).

**Fig. 4:**
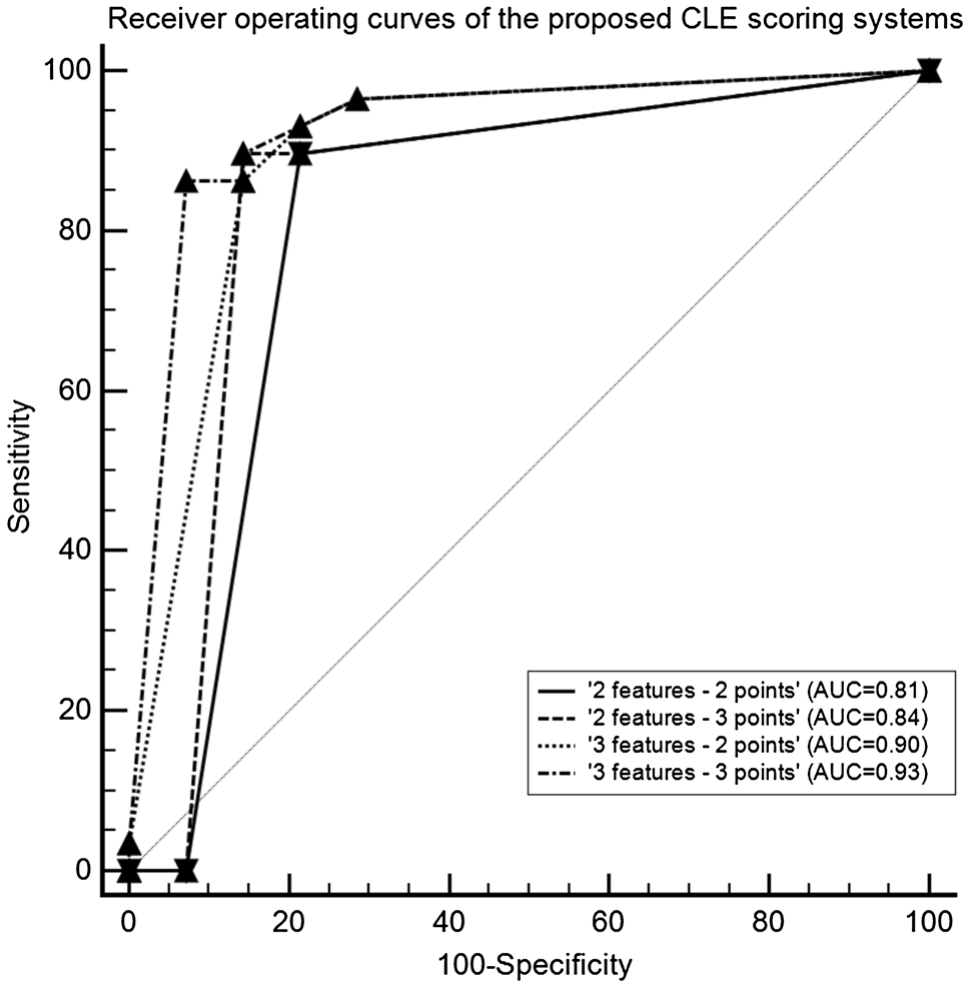
Receiver operating curves of the different CLE-based scoring systems

**Fig. 5:**
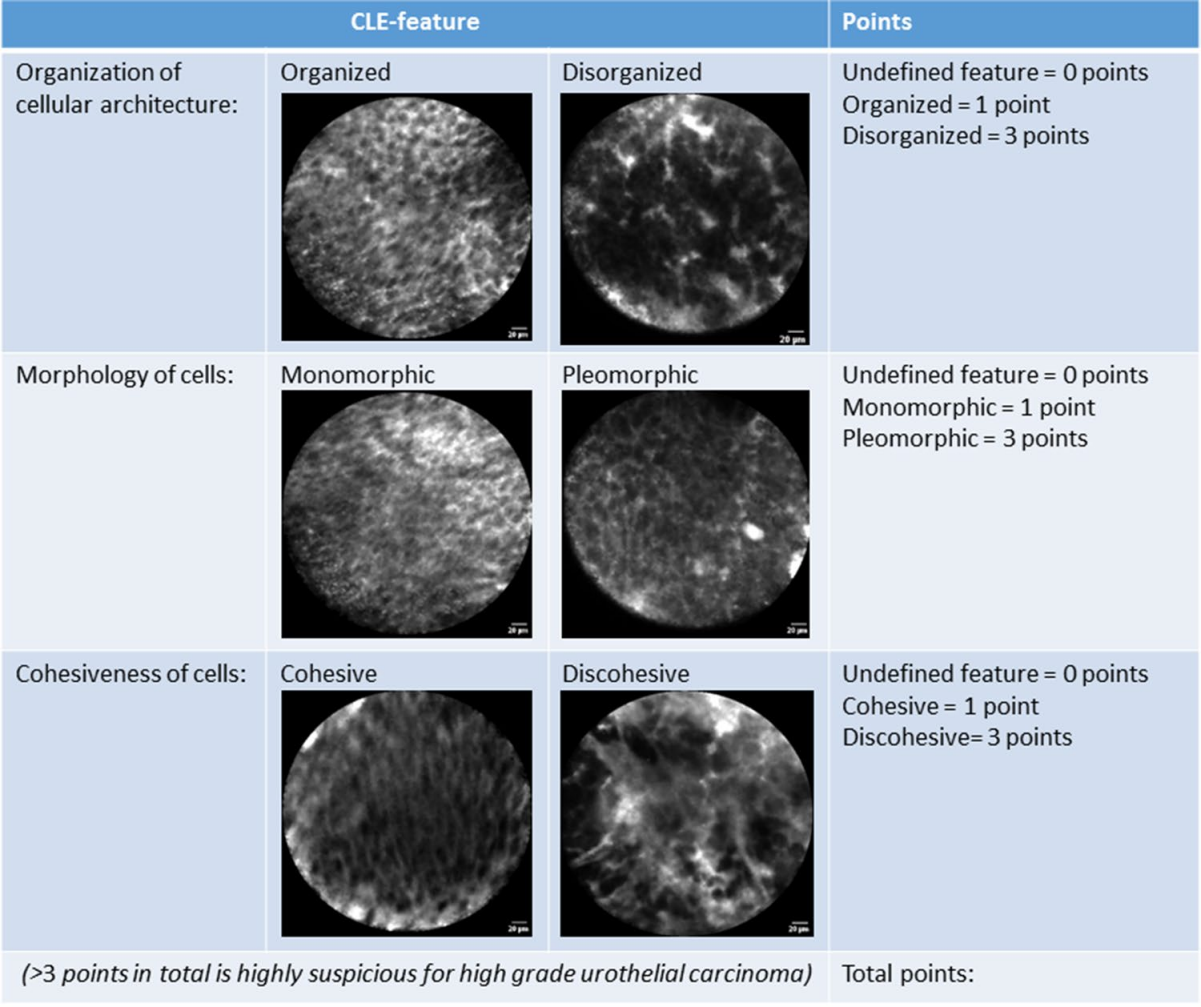
CLE-based scoring system for grading of papillary UTUC

The ROCs of each scoring system are presented in Fig. 4. DeLong testing resulted in a statistically significant difference for the pairwise comparison of the AUCs of the ‘3 features—2 points’ and the ‘2 features—2 points’ scoring system only (*p* = 0.045). The individual AUC of each scoring system with the optimal cutoff and corresponding sensitivity and specificity are presented in Table 3. The ‘3 features—3 points’ CLE-based scoring system, as illustrated in Fig. 5, yielded the highest diagnostic ability.

**Table 3:** Overview of the diagnostic abilities of the proposed CLE-based scoring systems

CLE-based scoring system	Area under the curve [CI]	Significance level of the AUC	Cutoff value for low-grade UTUC	Sensitivity at the cutoff	Specificity at the cutoff
‘2 criteria—2 points’	0.81 [0.66–0.91]	< 0.001	≤ 2	90 [73–98]	79 [49–95]
‘2 criteria—3 points’	0.84 [0.70–0.94]	< 0.001	≤ 2	90 [73–98]	86 [57–98]
‘3 criteria—2 points’	0.90 [0.77–0.97]	0.045	≤ 3	86 [68–96]	86 [57–98]
‘3 criteria—3 points’	0.93 [0.81–0.99]	< 0.001	≤ 3	86 [68–96]	93 [66–100]

*AUC* area under the curve, *CI* confidence interval, *CLE* confocal laser endomicroscopy, *UTUC* upper tract urothelial carcinoma

## Discussion

With this study, we confirm that the previously reported CLE criteria for UCB are also applicable for ureteroscopic CLE images of papillary UTUC. However, the visual appearance and the prevalence of the CLE criteria differ from UCB. Although preliminary, the assessment of CLE criteria allows for accurate identification of the histopathologic grade in papillary UTUC. The most prevalent CLE criteria with also the highest diagnostic potential for UTUC grading are cellular organization, morphology and cohesiveness of cells.

The difference in visual appearance and prevalence of the CLE criteria in UTUC compared to UCB can be explained by the different optical systems of the ureteroscopic CLE probe. The decreased ability to discriminate between two objects (inferior resolution) and the greater superimposition of cellular structures (larger depth of the confocal plane) results in inferior definition and sharpness of the ureteroscopic CLE images [16]. Consequently, cell borders were not clearly defined and, therefore, not assessable in the majority of low-grade and high-grade UTUCs. As a result, the diagnostic potential of this criterion for tumor grading, as described by Liem et al. [14] for UCB, could not be confirmed for UTUC. Similarly, the state of cellular polarity was often undefined in CLE images of UTUC. The papillary configuration and fibrovascular stalks were identifiable in almost all the ureteroscopic CLE recordings. Since these criteria are by definition present in papillary UTUC, they do not aid UTUC grading.

Chang et al. [10] suggested that tortuous vessels are characteristic for high-grade UC. In our study, the definition of tortuous vessels was deemed subjective and could not be identified accurately in ureteroscopic CLE images.

The inter-rater agreement for the individual CLE criteria was acceptable except for cellular cohesiveness. The inter-rater percentage agreement of this criterion was slightly below the threshold for acceptable agreement. The results for the inter-rater agreement are in line with the literature [12, 14]. In addition, the estimates of the sensitivity and specificity for CLE-based grading are in line with the results by Breda et al. [12]. As such, the proposed CLE criteria enable reproducible and accurate assessment of the UTUC grade.

The proposed scoring system based on cellular organization, morphology and cohesiveness with an allocation of 3 points for high-grade features resulted in the highest sensitivity and specificity for UTUC grading. However, the reproducibility of cellular cohesiveness was below the threshold for acceptable agreement, which could limit the diagnostic ability of the scoring system. The scoring system based on only cellular organization and morphology with an allocation of 3 points for high-grade features resulted in a very similar ROC and AUC. Yet, despite a slightly higher sensitivity, the scoring system based on two criteria resulted in a lower specificity than the scoring system based on three criteria. Reducing the number of CLE criteria and quantifying the significance of CLE features for tumor grading can contribute towards simplification and standardization of CLE image assessment. This would enhance the clinical applicability of CLE for intra-operative grading of UTUC. Nevertheless, external validation and comparison of both scoring systems is required in future studies.

In the present study, the non-diagnostic yield of ureteroscopic biopsies (8/70) was within the range of reported rates (10–20%) [4–6, 8]. The non-diagnostic yield of CLE-based grading (10/53), however, was higher than reported in the study by Breda et al. [12]. This difference could be attributed to the fact that the raters of Breda et al. also had knowledge of the ureteroscopic appearance of the imaged tumors. Another important aspect of CLE image quality is the application of fluorescein. Investigations of the pharmacokinetics of fluorescein with regard to the urothelium may help to optimize the procedural protocol. Furthermore, the durability of the CLE probe might also be a point of concern as the image quality seemed to deteriorate with cumulative probe use. In addition, the anatomical site of the tumor might influence the CLE image quality. Of the ten non-diagnostic CLE recordings, four tumors were located in the lower pole and three in a stenotic ureter. Next, the learning curve of CLE application and interpretation may influence the diagnostic yield. The surgeons and raters of the current study, however, were already familiar with the technique. Familiarization with application and interpretation of CLE was achieved within a small number of cases prior to the present study [14, 18].

In theory, confocal laser endomicroscopy allows for in vivo assessment of the complete tumor and therewith may avoid undergrading with regard to intra-tumoral heterogeneity or sampling error [5, 21, 22]. Moreover, CLE-based grading of papillary UTUC in vivo may allow for accurate intra-operative risk stratification and hence facilitation of immediate treatment selection. This implementation could lead to a reduction in the number of subsequent URS, surgery time and health-care costs.

The next step in the development of CLE as a tool for real-time tumor grading requires a powered analysis of its diagnostic accuracy during ureteroscopy, preferably in combination with a validation of the proposed scoring system. Additionally, decision curve analysis may be a valuable tool to evaluate the net benefit of CLE for UTUC diagnosis [23]. More data on CLE for UTUC grading is also needed for the development of convolutional neural networks for computer-aided image assessment. Due to the low incidence of UTUC, a joint multicenter approach is required to achieve powered studies for such analyses within a reasonable timeframe.

## Limitations

First, the histopathologic findings of co-localized ureteroscopic biopsies were used as the reference standard for comparison. The histopathologic grade of ureteroscopic biopsies may not be accurate in comparison to the histopathology of surgical resections due to possible grade heterogeneity, sampling error or subjectivity of the histopathologic assessment [4, 5, 21, 22]. On the other hand, biopsies allow for superior macroscopic co-localization of the histopathologic assessed tissue and the imaged region with the index test than resection specimens. Yet more importantly, relying on the histopathologic grade of ureteroscopic biopsies did not allow for a direct comparison of the diagnostic yield and accuracy between CLE imaging and biopsies. Studies of comparative accuracy are required to identify the potential role of CLE for the current diagnostic pathway [24].

The proposed scoring system was based on univariate analysis. The accuracy of the scoring system could be improved with multivariate statistics and an increased sample size [25]. Moreover, the proposed scoring system requires validation.

Next, the histopathologic assessment of biopsies was performed by a single uropathologist. While this single-rater approach avoided inter-rater variability, the most accurate histopathologic grading would result from an expert panel consensus [26].

Besides grading of UC, CLE may also be used as a diagnostic tool for the identification of carcinoma in situ amongst flat lesions [12–14]. This was, however, not addressed in the current study because the assessment of flat lesions should be regarded as a separate diagnostic algorithm with a different clinical implication than grading of papillary UTUC. The potential of CLE for flat lesions remains to be investigated.

A technical limitation of CLE is the requirement of a fluorescent contrast agent. Besides adding an extra preparation step, the ureteroscopic vision after fluorescence application may be hampered. The vision can be improved by flushing saline through the ureteroscope, but is time consuming and should be minimalized to avoid high intra-renal pressures.

## Conclusion

CLE allows for accurate grading of papillary UTUC with the previously described CLE criteria for urothelial carcinoma. The most prevalent and discriminating CLE criteria in papillary UTUC are cellular organization, morphology and cohesiveness. The proposed scoring system based on these criteria for UTUC grading may allow for a more quantifiable and simplified approach at a similar diagnostic accuracy. External validation of the proposed scoring system is required.

## Abbreviations

AUC: Area under the curve
CI: Confidence interval
CLE: Confocal laser endomicroscopy
NPV: Negative predictive value
PPV: Positive predictive value
ROC: Receiver operating curve
SD: Standard deviation
UC: Urothelial carcinoma
UCB: Urothelial carcinoma of the bladder
UTUC: Upper tract urothelial carcinoma

## References

[1] Seisen T, Peyronnet B, Dominguez-Escrig JL (2016). Oncologic outcomes of kidney-sparing surgery versus radical nephroureterectomy for upper tract urothelial carcinoma: a systematic review by the EAU non-muscle invasive bladder cancer guidelines panel. Eur Urol.

[2] Rouprêt M, Babjuk M, Compérat E (2017). European Association of Urology Guidelines on Upper Urinary Tract Urothelial Carcinoma: 2017 Update. Eur Urol.

[3] Rouprêt M, Colin P, Yates DR (2014). A new proposal to risk stratify urothelial carcinomas of the upper urinary tract (UTUCs) in a predefinitive treatment setting: low-risk versus high-risk UTUCs. Eur Urol.

[4] Margolin EJ, Matulay JT, Li G (2018). Discordance between ureteroscopic biopsy and final pathology for upper tract urothelial carcinoma. J Urol.

[5] Straub J, Strittmatter F, Karl A, Stief CG, Tritschler S (2013). Ureterorenoscopic biopsy and urinary cytology according to the 2004 WHO classification underestimate tumor grading in upper urinary tract urothelial carcinoma. Urol Oncol.

[6] Wang JK, Tollefson MK, Krambeck AE, Trost LW, Thompson RH (2012). High rate of pathologic upgrading at nephroureterectomy for upper tract urothelial carcinoma. Urology.

[7] Smith AK, Stephenson AJ, Lane BR (2011). Inadequacy of biopsy for diagnosis of upper tract urothelial carcinoma: implications for conservative management. Urology.

[8] Keeley FX, Kulp DA, Bibbo M, McCue PA, Bagley DH (1997). Diagnostic accuracy of ureteroscopic biopsy in upper tract transitional cell carcinoma. J Urol.

[9] Bui D, Mach KE, Zlatev DV, Rouse RV, Leppert JT, Liao JC (2015). A pilot study of in vivo confocal laser endomicroscopy of upper tract urothelial carcinoma. J Endourol.

[10] Chang TC, Liu JJ, Hsiao ST (2013). Interobserver agreement of confocal laser endomicroscopy for bladder cancer. J Endourol.

[11] Liu JJ, Hsiao ST, Pan Y (2011). Real time diagnosis of bladder cancer with probe-based confocal laser endomicroscopy: a prospective diagnostic accuracy study. J Endourol.

[12] Breda A, Territo A, Guttilla A (2017). Correlation between confocal laser endomicroscopy (Cellvizio^®^) and histological grading of upper tract urothelial carcinoma: a step forward for a better selection of patients suitable for conservative management. Eur Urol Focus.

[13] Villa L, Cloutier J, Cote JF, Salonia A, Montorsi F, Traxer O (2016). Confocal laser endomicroscopy in the management of endoscopically treated upper urinary tract urothelial cell carcinoma (UTUC): preliminary data. Eur Urol Suppl..

[14] Liem EIML, Freund JE, Savci-Heijink CD (2018). Validation of confocal laser endomicroscopy features of bladder cancer : the next step towards real-time histologic grading. Eur Urol Focus.

[15] Eble JN, Sauter G, Epstein JI, Sesterhenn IA (2004). Pathology and genetics of tumours of the urinary system and male genital organs. World Heal Organ Classif Tumours.

[16] Adams W, Wu K, Liu JJ, Hsiao ST, Jensen KC, Liao JC (2011). Comparison of 2.6- and 1.4-mm imaging probes for confocal laser endomicroscopy of the urinary tract. J Endourol..

[17] McCulloch P, Altman DG, Campbell WB (2009). No surgical innovation without evaluation: the IDEAL recommendations. Lancet.

[18] Liem EIML, Freund JE, Baard J (2018). Confocal laser endomicroscopy for the diagnosis of urothelial carcinoma in the bladder and the upper urinary tract: Protocols for two prospective explorative studies. JMIR Res Protoc.

[19] Freund JE, Liem EI, Baard J (2018). Confocal laser endomicroscopy for the diagnosis of urothelial carcinoma in the bladder and the upper urinary tract. Videourology.

[20] McHugh ML (2012). Interrater reliability: the kappa statistic. Biochem Med..

[21] Cheng L, Neumann RM, Nehra A, Spotts BE, Weaver AL, Bostwick DG (2000). Cancer heterogeneity and its biologic implications in the grading of urothelial carcinoma. Cancer.

[22] Höglund M (2015). Heterogeneous challenges for urologic cancers. Eur Urol.

[23] Vickers A, Elkin E (2006). Decision curve analysis: a novel method for evaluating prediction models. Med Decis Mak..

[24] Bossuyt PM, Irwig L, Craig J (2006). Comparative accuracy: assessing new tests against existing diagnostic pathways. Br Med J.

[25] Grobman WA, Stamilio DM (2006). Methods of clinical prediction. Am J Obstet Gynecol.

[26] May M, Brookman-Amissah S, Roigas J (2010). Prognostic accuracy of individual uropathologists in noninvasive urinary bladder carcinoma: a multicentre study comparing the 1973 and 2004 World Health Organisation Classifications. Eur Urol.

